# Pheromones and Other Semiochemicals for Monitoring Rare and Endangered Species

**DOI:** 10.1007/s10886-016-0753-4

**Published:** 2016-09-13

**Authors:** Mattias C. Larsson

**Affiliations:** Department of Plant Protection Biology, Swedish University of Agricultural Sciences, P.O. Box 102, 230 53 Alnarp, Sweden

**Keywords:** Biodiversity, Conservation biology, Landscape ecology, Monitoring, Pheromones, Dispersal, Population

## Abstract

As global biodiversity declines, biodiversity and conservation have become ever more important research topics. Research in chemical ecology for conservation purposes has not adapted to address this need. During the last 10–15 years, only a few insect pheromones have been developed for biodiversity and conservation studies, including the identification and application of pheromones specifically for population monitoring. These investigations, supplemented with our knowledge from decades of studying pest insects, demonstrate that monitoring with pheromones and other semiochemicals can be applied widely for conservation of rare and threatened insects. Here, I summarize ongoing conservation research, and outline potential applications of chemical ecology and pheromone-based monitoring to studies of insect biodiversity and conservation research. Such applications include monitoring of insect population dynamics and distribution changes, including delineation of current ranges, the tracking of range expansions and contractions, and determination of their underlying causes. Sensitive and selective monitoring systems can further elucidate the importance of insect dispersal and landscape movements for conservation. Pheromone-based monitoring of indicator species will also be useful in identifying biodiversity hotspots, and in characterizing general changes in biodiversity in response to landscape, climatic, or other environmental changes.

## Introduction

Global agricultural and forestry practices frequently are in direct conflict with biodiversity and associated ecosystem services, and thus conservation issues are an increasingly important focus of research (Grove 2002; Kleijn et al. 2009; Ricketts et al. 2008). Measures to halt the decline of biodiversity often have proven ineffective (Batary et al. 2015; Butchart et al. 2010), which increases the need for evidence-based conservation strategies. Insects represent the most diverse group of animals, and include high numbers and proportions of threatened species (Brooks et al. 2012; Conrad et al. 2006). They also constitute essential components of food webs in terrestrial and aquatic ecosystems, and provide important ecosystem services such as pollination, pest control, and recycling of biomass. However, monitoring their distribution and abundance is a formidable task that constitutes an important barrier to evidence-based conservation efforts.

Ever since the first characterization of a sex pheromone in the silk moth *Bombyx mori* (Butenandt et al. 1959), identification and application of insect pheromones have focused on management of insect pests (Smart et al. 2014; Witzgall et al. 2010). Pheromones have been used sporadically by collectors and conservationists in their search for rare and cryptic species, for example by using live females to attract males (Mari-Mena et al. 2016), or by utilizing single synthetic pheromone components or partial pheromone blends (Buda et al. 1993). During the last 10–15 years, however, increasing attention has been directed towards exploiting the powerful attraction of insect pheromones as monitoring tools in biodiversity and conservation research. The first insect pheromone identified specifically as a tool for conservation was (*R*)-γ-decalactone, the sex or aggregation pheromone of the threatened scarab beetle *Osmoderma eremita* (Larsson et al. 2003). Since then, several other pheromones have been identified with explicit or implicit applicability for insect conservation (Barbour et al. 2011; Gago et al. 2013; Konig et al. 2016; Millar et al. 2010; Ray et al. 2012, 2014; Tolasch et al. 2007, 2013; Yan et al. 2015), with additional semiochemicals being the target of ongoing studies (Harvey et al. 2011).

Pheromones may be more suitable for conservation monitoring than for their originally envisioned purpose of pest management, simply because the bar for usefulness is much lower. Pheromones excel at providing reasonably reliable indicators of the presence of a target species at very low population density, where other monitoring methods fall short. Whereas there is probably less commercial value in conservation compared to pest management, public spending on biodiversity and conservation is nevertheless considerable and growing. In addition, commercial enterprises in the agricultural and forestry sectors, or certification programs, may also benefit from evidence-based demonstrations of concrete results from their environmental policies. There is, therefore, great societal value and a potential market for research and development in monitoring systems specifically for use in conservation efforts.

Given the great potential rewards from pheromone monitoring within insect biodiversity and conservation research, it is remarkable that there have been so few practical applications of pheromones within this field. The reason for the persistent focus on pest systems among chemical ecologists may be largely a result of the difficulties in obtaining competitive funding for identification of pheromones of non-pest species, which has created a considerable barrier to the development of model systems to demonstrate the benefits of these techniques for conservation. Pest management also constitutes a diametrically opposing point of view than that which conservationists bring to their respective model systems, and this may have reinforced a lack of collaborative efforts. The aim of the present paper is to encourage cross-talk by informing conservation biologists about the possibilities and practical aspects of using pheromones and other semiochemicals as tools, and conversely, to inform chemical ecologists about the practical developments needed in order to answer critical questions within conservation biology. Here, I summarize published and some as yet unpublished research on semiochemicals of species of general interest for biodiversity and conservation research, with an emphasis on systems in which pheromones can be exploited to substantial benefit. I also address how these systems have been utilized to answer questions regarding the ecology and interactions of model species, as well as the potential for future applications, with knowledge drawn from pest systems where pheromones have been applied to answer similar questions for decades. The material presented on conservation management has a European bias because conservation management as outlined here appears to be disproportionately practiced in Europe. This may reflect traditions from long-term historical integration of natural and cultivated ecosystems, which have been heavily influenced by human activity in Europe, combined with the large-scale structure of European conservation politics.

## Usefulness of Different Semiochemicals for Conservation Monitoring

Assessing the distribution, abundance, and population trends of individual species, communities, and whole ecosystems constitutes a core aspect of general ecology, biodiversity, and conservation research. One absolutely fundamental task for conservation is to estimate extinction risk, summarized as red list status (Anonymous 2012), and to identify the key factors responsible for the decline of species in order to halt or reverse negative trends (Anonymous 2012; Mace et al. 2008; Miller et al. 2007). However, the scattered information available regarding past and present distribution and abundance of insects makes the evaluation procedure a mixture of educated guesswork and evidence-based science (Jeppsson et al. 2010; Lindhe et al. 2010).

Systematic surveys of insects, based on broad-spectrum, stochastic collection methods such as light traps, window traps, pan traps, or pitfall traps (Bates et al. 2014; Driscoll 2010; Jansson 2009; Samways et al. 2010) provide an overview of general trends, but their ability to provide fine-grained information about individual species is limited (Driscoll 2010), and often requires intense efforts that combine several methods (Ranius and Jansson 2002). Traps based on random encounters also have a major sorting problem, i.e., reliable identification of the target insect among all other insects caught. Low probability of detection constitutes another general problem in biodiversity research, and may lead to both over- and underestimations of the true distributions and extinction risks (Kery and Schmidt 2008).

**Fig. 1:**
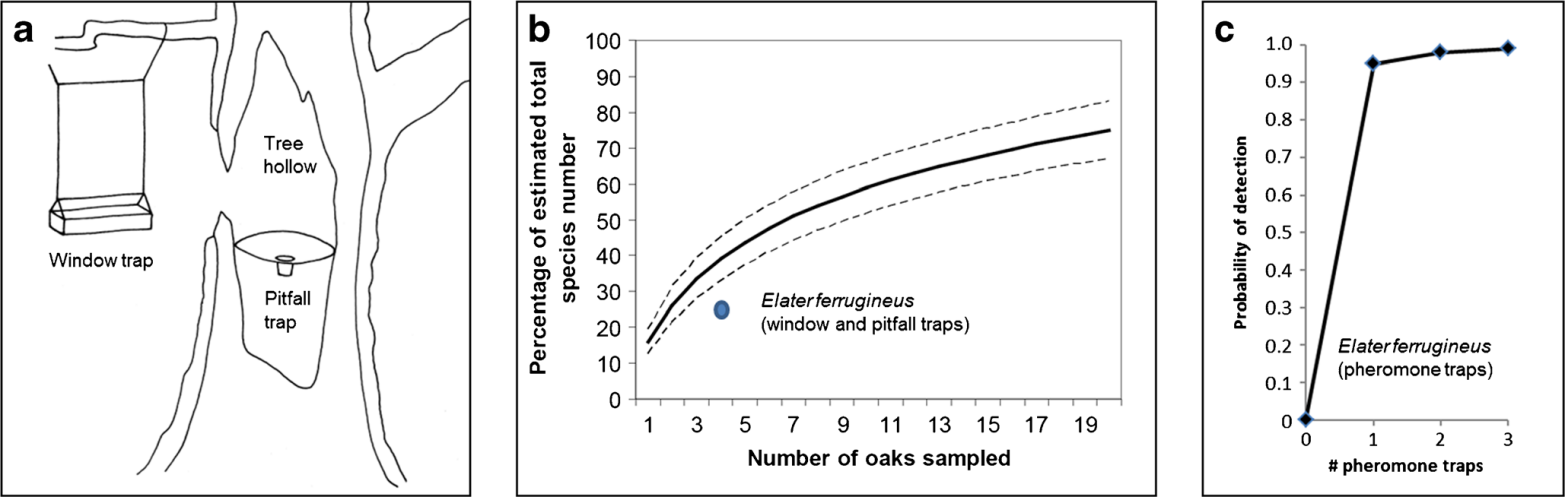
An example of the increased probability of detection of insect populations using pheromone-based traps, compared to traditional window and pitfall trapping. **a**. Jansson (2009) performed a large-scale study of saproxylic insects in hollow oaks at a large number of localities. Each oak was sampled with two traps over a whole season: one window trap placed in front of the largest entrance, and one pitfall trap buried in the wood mold material inside the hollow. **b**. At one site 20 different oaks were sampled, which allowed an estimate of the proportion of all saproxylic insects detected at a locality, based on the number of oaks sampled. The average estimate for all species is displayed by a rarefaction curve with 95% confidence intervals, showing that large sampling efforts are needed: approximately 8 oaks need to be sampled to detect 50% of all saproxylic insect species at the locality (Jansson 2009). The dot under the curve represents the corresponding detection probability per locality (approximately 26%) for the rust red click beetle *Elater ferrugineus* sampled with an effort of four oaks per locality at many different localities where the species was known to be present (Andersson 2012; Andersson et al. 2014). **c**. Representation of the approximate corresponding probability of detecting a species such as the rust red click beetle *Elater ferrugineus* at the same sites and additional experimental sites, in relation to the number of pheromone traps used per site (Andersson 2012; Andersson et al. 2014; Svensson et al. 2012 and unpublished data). The curve is more of a general conceptual illustration than actual data; in reality a single trap had a 100% detection rate at all known localities for *E. ferrugineus* in our studies, but could presumably fail occasionally at extremely low densities. Images **a** and **b** modified from Nicklas Jansson (with permission)

For a large number of insect species of conservation interest, monitoring with pheromones or other semiochemicals has the potential to completely reverse this situation. The attractiveness of many insect pheromones could facilitate monitoring at an unprecedented spatiotemporal resolution with great efficiency, while achieving a detection probability near 1.0 even for relatively sparse populations of insects that would otherwise be difficult, or virtually impossible, to detect (Fig. 1, and see below).

Ideal monitoring systems for conservation have properties similar to those that would apply for pest management systems, specifically, highly efficient long-range attractants that are easy to exploit for monitoring purposes, including large-scale production of synthetic semiochemicals. Identification of novel semiochemicals may be complicated by logistical problems in obtaining unmated adults, particuarly for rare and threatened species. To aid in the collection of enough pheromone to identify, the insects’ pheromone production can in principle be enhanced by hormonal treatment, even in mated individuals (Dickens et al. 2002; Groot et al. 2005).

With a very rough generalization, canonical sex attractant pheromones (which attract one sex only) typical of moths and many other insect groups are released by females, with males as the responding sex. Females release pheromones in small quantities, typically in nanogram or less quantities, exclusively or mainly while still unmated, and the pheromones elicit obvious and rapid responses from males. The small quantities of sex pheromones typically released by female insects frequently constitute a considerable barrier to identification even for highly abundant pest insects, because it can be difficult to find the pheromone in crude extracts, unless techniques like coupled gas chromatography-electroantennography are used. It can be even more difficult and/or time consuming to obtain enough of the pure compound(s) to identify.

In contrast, pheromones released by male insects often are emitted in larger quantities (micrograms per male or more), during longer periods of the male’s life if he is able to mate multiple times, and they often work as sex-aggregation pheromones (attracting both sexes), though rarely with the same efficiency as female-released sex pheromones (Larsson et al. 2003; Schlyter and Birgersson 1999). The relatively large quantities released by males may facilitate identification of these sex aggregation pheromones. However, because of the relatively high release rates, lures for many male-produced sex-aggregation pheromones must be loaded with correspondingly large doses of pheromone (often >100 mg/lure) in order to provide a lure capable of releasing several milligrams per day for periods of several weeks. Thus, these pheromones can be used only for practical applications if the pheromones can be synthesized cheaply and in large scale.

Almost all published pheromones of explicit conservation interest have been female-produced sex attractant pheromones, including those of the Spanish moon moth *Graellsia isabellae* (Millar et al. 2010) and other moths (Gago et al. 2013; Yan et al. 2015), the rust red click beetle *Elater ferrugineus* (Svensson et al. 2012; Tolasch et al. 2007) and related species (Konig et al. 2016; Tolasch et al. 2013), and longhorn beetles in the genera *Prionus* (Barbour et al. 2011), *Tragosoma* (Ray et al. 2012), and *Desmocerus* (Ray et al. 2014). The only exception so far is the male-produced sex-aggregation pheromones of scarab beetles in the genus *Osmoderma* (Larsson et al. 2003; Svensson et al. 2009; Zauli et al. 2016). Identifications of several other sex-aggregation pheromones of longhorn beetles of conservation concern are ongoing (for an overview of potentially interesting model genera see Hanks and Millar 2016).

Other semiochemicals useful for conservation monitoring include various kairomones, i.e., attractants utilized by insects to locate prey, hosts, or food. Generally, these compounds attract a broader spectrum of species that exploit similar resources, and are considerably less attractive than pheromones, but may nevertheless be useful for monitoring. The complex of saproxylic and xylophagous bark beetles, click beetles, longhorn beetles, and other insects that are attracted to terpenoids and alcohols from various host trees constitute a classical example (Gandhi et al. 2009; Miller and Rabaglia 2009). Other kairomonal attractants useful for practical conservation or pest management, either used as synthetic baits or natural sources with potential for developing synthetic replacements, may include carrion (Creighton and Schnell 1998; Hanski et al. 2007), dung (Hanski et al. 2007), fruit and fermentation baits (Benedick et al. 2006; Jonason et al. 2013), and floral/plant volatiles (Bengtsson et al. 2009; Gregg et al. 2010; Ladd and McGovern 1980). The *Osmoderma* spp. pheromone also constitutes a kairomone that attracts female *E. ferrugineus* (predators of larval *Osmoderma* spp.), and has been used for monitoring *E. ferrugineus* females in the field (Larsson and Svensson 2009, 2011; Svensson et al. 2004; Zauli et al. 2014).

## Model Systems and Their Usefulness for Conservation Monitoring

When selecting insect model systems for monitoring from a perspective of biodiversity and conservation, it is important to prioritize spending of limited resources. It is worth emphasizing, however, that when individual insect species are given special priority for conservation, for example within specific action plans or management schemes for critically endangered species, it will almost always be a good investment to develop an effective monitoring system based on pheromones. Any management program intended to safeguard the long-term persistence of a species will need to allocate resources for continued evaluation of its success. An effective pheromone monitoring system will drastically improve the cost-benefit ratio of this process.

In most cases, individual species are not the main focus of conservation efforts. Instead, the preservation of entire habitat elements or whole communities at the larger landscape or regional scale is usually the goal. Such large-scale conservation schemes affect hundreds or thousands of species, from vascular plants to invertebrates and vertebrates. In these cases, monitoring efforts are aimed at evaluating the overall processes affecting gain or loss of biodiversity rather than monitoring the fate of individual species (Batary et al. 2015). Offering monitoring systems for a handful of insect species may be a hard sell, unless they actually advance our understanding of these general processes beyond that offered by already existing systems. In this context, in order for monitoring systems to make a difference in conservation efforts, one should consider carefully which model systems are worth developing. Various types of natural and cultural ecosystems differ markedly with regards to the potential model species they offer, as well as their applicability to already established frameworks of conservation schemes. Two general comparisons between different systems of conservation concern may be useful in illustrating this point: cultivated agricultural and semi-natural ecosystems, as compared to forest ecosystems with insects associated with dead and decaying wood, respectively.

Cultivated or semi-natural ecosystems associated with or immediately affected by agricultural production and livestock farming are of immediate concern for conservation, and considerable resources have been diverted to their sustainable use, including preservation of biodiversity and ecosystem services. However, their short-term potential for establishing competitive model systems for pheromone monitoring of biodiversity may be limited. There are already well established insect model indicator groups for monitoring landscape-wide biodiversity within these agroecosystems, including representatives for important ecosystem services such as pollination, and natural enemies of agricultural pests. These indicator groups include butterflies, moths, bees, hoverflies, and carabid beetles (Bommarco et al. 2012; Brooks et al. 2012; Ekroos et al. 2013; Geiger et al. 2010; Kremen and M'Gonigle 2015). Most of these groups of insects apparently do not use long-range pheromones that could be exploited for monitoring, they are often visually conspicuous, and/or they can be sampled reasonably well through alternative means such as manual surveys or unbaited pitfall or pan traps. Of these groups, only moths could be immediately exploited as targets for pheromone monitoring, including the diurnal burnet moths (family Zygaenidae), for which some pheromones are already available for European species (El-Sayed 2012; Priesner et al. 1984; Subchev 2014). In particular, as with many nectar-feeding insects, these moths have been declining and could be useful biodiversity indicators (Sarin and Bergman 2010).

Developing standardized floral or fermentation mimics for monitoring floral visitors (Gregg et al. 2010), or specialized lures for other groups like dung beetles, could significantly expand the applicability of standardized monitoring with semiochemical attractants in these landscape systems. Provided that the right species are targeted, there could be excellent potential to develop monitoring systems that would provide information about the effects of landscape change at much finer-grained scales than those provided by large-scale monitoring schemes with light traps (Bates et al. 2014). There are other potential groups of model species for habitats within these landscape systems, such as root-feeding click beetles associated with natural or semi-natural grasslands. Unlike moths, these insects are not part of already established indicator groups and would therefore need general evaluation of their potential to reflect different processes associated with landscape change. Indeed, they may have better potential to illuminate relevant aspects of grassland ecosystems and their associated communities than other more well-studied insect groups.

In contrast to the indicator species in cultivated or semi-natural ecosystems associated with agriculture or livestock farming, insects from saproxylic (dead-wood-associated) or xylophagous (wood-feeding) communities in forest or woodland ecosystems may offer a wider selection of tractable model species for monitoring with pheromones, in addition to already established attraction to host tree kairomones. Some groups of forest insects that are already attracting considerable interest as model systems for conservation are known to use attractant pheromones, such as longhorn beetles (Hanks and Millar 2016), click beetles (Toth 2013), scarabaeoids (Vuts et al. 2014), and several groups of moths, although to date, only a few pheromones have been identified for species of conservation interest (Barbour et al. 2011; Konig et al. 2016; Larsson et al. 2003; Ray et al. 2012, 2014; Svensson et al. 2012; Tolasch et al. 2007). There also is a diverse community of additional saproxylic insects that are known or could be expected to use pheromone communication, including beetles in many other families (El-Sayed 2012; Francke and Dettner 2005) and also different species of saproxylic dipterans, including some cranefly species that exhibit obvious antennal sexual dimorphism. Because their chemical ecology is poorly known, it is an open question as to whether these insect groups might be amenable to development of their pheromones for practical uses. Many saproxylic insects are frequently trapped in various monitoring systems that are directed towards forest insects, and as by-catch in semiochemical-baited traps for forest pest insects. Their general distribution and indicator potential are well known. Thus, there is great potential for integrating novel pheromone monitoring methods into existing conservation programs, and by doing so, providing fine-grained spatiotemporal information to complement existing distribution records.

With respect to the logistics of obtaining specimens for pheromone identification, some guilds of saproxylic insects can be obtained (with some effort) as whole communities, by sampling dead wood from specific tree species at the right stage of decomposition. These insects often can be found aggregating as larvae in relatively high densities in decaying logs, which may be brought in during the winter season for forcing the emergence of adults in the laboratory by warming the logs. Moreover, we often have had good success with controlled inoculation of substrates with whole groups of species by placing logs at strategic locations and allowing them to become infested naturally.

## Estimating Change: Distributions and Population Sizes

The most basic aspects of population ecology and conservation concern whether populations change their geographical distribution and population sizes over time. Due to the aforementioned difficulties with obtaining good quality data even on presence and absence over time, it can be a serious challenge to demonstrate evidence-based changes in populations for a large fraction of insect species. Metapopulation dynamics, which deals with long-term persistence of populations in scattered habitat fragments, is preferentially studied in a limited selection of insects such as highly visible butterflies and bees, for which true presence-absence patterns can be established with some accuracy (Franzén and Nilsson 2009; Ojanen et al. 2013). For many other insect species, significant uncertainties regarding detection of their presence or absence would mean that most of the dynamic changes observed could be attributable to noise in detection, thus making questions about metapopulation dynamics virtually unanswerable.

Judging from available data from a few conservation species and a plethora of pest species, pheromone traps represent a significant improvement on almost any other method of monitoring. The efficiency of a trap can be represented by a function describing the proportion of individuals caught at different distances (Byers et al. 1989; Miller et al. 2010, 2015; Schlyter 1992; Turchin and Odendaal 1996). Trials may be performed either as recaptures in traps at a single central position from a single or several release points, or with a matrix of traps in different directions around a single release point. One important result from these trials is that, despite their high attraction, pheromone traps rarely capture most of the available insects at a distance beyond a few tens of meters away from a trap. Recapture rates for pheromone traps that target different insect taxa, such as moths, various groups of beetles, and sawflies, often range from around 10 to 30-40% (Kishita et al. 2003; Larsson and Svensson 2009; Maki et al. 2011; Östrand et al. 2001; Weslien and Lindelow 1990), although they may reach over 90% (Zhang and Schlyter 1996).

**Fig. 2:**
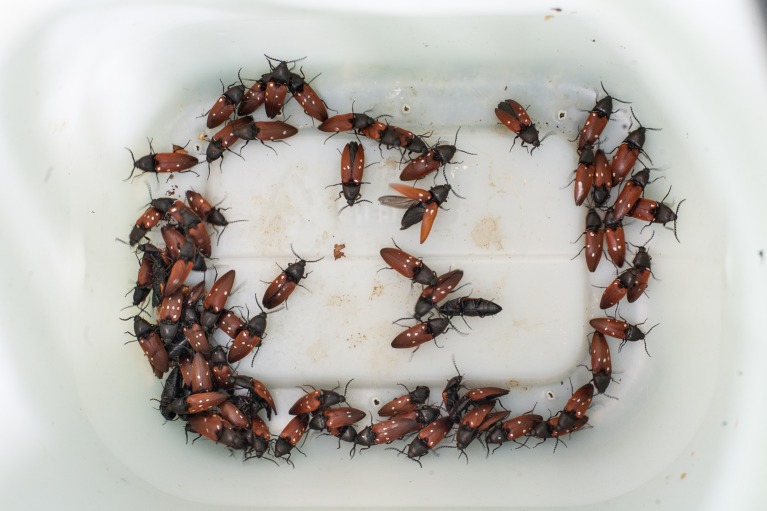
The formidable attraction potential of sex pheromones, illustrated by a single two-day catch of male rust red click beetles *Elater ferrugineus* at Hallands Väderö in southern Sweden. Before the use of pheromone-baited traps for *E. ferrugineus*, no entomologists would likely have encountered this number of adult specimens during a life-time. Each specimen in the trap has been individually marked before release, for the purpose of recapture studies. Photo: Benjamin Forsmark

At the population level, any trapping system that can catch 10-30% of the individuals in the vicinity of the trap will be highly efficient in detecting the presence of local populations, even at very low population and trap densities. There is, thus, every reason to believe that most sex or aggregation pheromone systems can provide accurate presence-absence estimates, with absence of catches indicating a true absence of a local population with near certainty. For example, the male-released aggregation/sex pheromone of hermit beetles (*Osmoderma* spp*.*) represents the least efficient pheromone trapping system developed for conservation monitoring, and is preferably used in combination with other methods such as pitfall trapping in individual trees (Andersson et al. 2014; Chiari et al. 2013b; Larsson and Svensson 2009; Zauli et al. 2014). Their sedentary nature inside tree hollows often renders only a small fraction of the beetle population available for pheromone trapping in Sweden (Ranius and Hedin 2001), whereas in warmer Mediterranean areas, beetles may be more mobile and so more easily caught (Chiari et al. 2013a). The sex pheromone system of *E. ferrugineus*, which lives in the same hollow tree habitat, constitutes a stark contrast to the *Osmoderma* pheromone, and represents one of the greatest transformations in detection ability based on pheromones. That is, *E. ferrugineus* is seldom observed or trapped in window or pitfall traps, leading to sparse observations of this species even among experienced entomologists before the identification of sex pheromone lures (Svensson et al. 2004; Tolasch et al. 2007). The first systematic trials using the sex pheromone revealed not only that *E. ferrugineus* can be present at high population densities at many sites, but that males often respond immediately to the pheromone, aggregating around baits in large numbers. Males are mobile and long-lived, with a high capture probability making the detection of local populations with pheromone-based monitoring methods a virtual certainty (Fig. 2) (Andersson et al. 2014; Kadej et al. 2015; Svensson et al. 2012; Tolasch et al. 2007; Zauli et al. 2014).

As a consequence, the pheromone of *E. ferrugineus* has been used successfully for large-scale landscape studies (Kadej et al. 2015; Musa et al. 2013; Oleksa et al. 2015) (J. Burman, D. Harvey and coworkers, unpublished data). Regional surveys also have been performed with the clearwing moth *Synanthedon vespiformis* (Burman et al. 2016). In addition, the author and his coworkers have unpublished data sets of red-listed saproxylic moths (family Tineidae) (J. Burman, G. P. Svensson, N. Ryrholm and coworkers), burnet moths (*Zygaena spp*.) (J. Burman and coworkers), and several species of longhorn beetles (I. Winde, M. Molander, and coworkers). These studies have demonstrated that information from pheromone-based trapping during a single field season generally can surpass the known information about most rare species several times over, and that existing records often provide an underestimation of their true distributions. Pheromones also have been used for systematic DNA sampling for phylogeographic studies of *E. ferrugineus* (Oleksa et al. 2015) and the Spanish moon moth *G. isabellae* (Mari-Mena et al. 2016). In parallel with monitoring for conservation purposes, monitoring systems with pheromones and other semiochemicals support the detection of invasive pest species at low densities (Liebhold et al. 2016), including monitoring their range expansion (Liebhold and Bascompte 2003; Suckling et al. 2014), and the effectiveness of eradication efforts (Kean and Suckling 2005; Kikkert et al. 2006; Lance and Gates 1994).

For semiochemicals other than pheromones, uncertainties of presence or absence have to be adjusted according to the estimated capture probabilities of the respective systems; as stated above, these generally are lower than detection by pheromone-baited traps (Benedick et al. 2006; Creighton and Schnell 1998; Jurzenski et al. 2014). Unless recapture studies are performed, estimating the true probability of detection for individual species may be difficult, but broad-spectrum captures may still reveal trends for whole communities (Hanski et al. 2007).

In addition to presence/absence, pheromone traps also can furnish quantitative measures of abundance, thus providing hard evidence for change in the form of increases or decreases in local populations. Standardized trap catches constitute the least complicated and most commonly used indicator of abundance (Blackshaw and Vernon 2006; Erbilgin et al. 2002; Gandhi et al. 2009), although with the important qualifier that many other factors apart from population density will affect the number of insects that are actually caught in a trap, with variation in relation to climate, weather conditions, and dispersal (Larsson and Svensson 2011; Zauli et al. 2014). Nevertheless, if traps are deployed under similar conditions, for example, simultaneously in areas in close proximity or with similar weather conditions, local abundance measures between sites likely will reflect local differences in population density (Collier et al. 2008). Mark-recapture functions that describe recapture probability distributions at different distances, as described above, allow estimates of absolute density based on captures in single traps (Miller et al. 2015). In pest management, pheromone-based monitoring is to a great extent focused on indications of abundance in relation to economic injury thresholds. Most of these studies deal with future predictions of abundance and/or damage levels based on host and pest phenology and weather conditions (Anderson et al. 2012; Damos and Savopoulou-Soultani 2010; Dömötör et al. 2007; Hayes et al. 2009; Mori et al. 2014). Nevertheless, there are a considerable number of studies that relate trap catches to abiotic conditions, which would constitute a platform for normalizing catches between different occasions (Williams et al. 2008). Without independent information regarding population density, however, it is difficult to differentiate between the effects of flight activity and population density on trap catches.

When determining abundance via sampling, estimates of absolute population density obtained by means of mark-recapture models constitute the gold standard. Absolute population sizes can be estimated as a simple function of the total catch and the proportion of recaptured individuals (Weslien and Lindelöw 1989). Keeping track of individuals over successive recapture events allows for more advanced statistical models, assuming either closed populations without migration or open population models that allow for migration or emergence/death of individuals (Ranius 2001; Tikkamäki and Komonen 2011). For rare and threatened insects, absolute population density is highly relevant information, not only for relating trap catches to population abundance, but also because population levels of some species actually may be so low that this in itself is a matter of concern. Nevertheless, their low probability of capture in traps, that rely only on random encounters with traps, renders this trapping method entirely unfeasible for many insects. Consequently, population estimates by mark-recapture studies that do not use pheromone-based methods are heavily skewed towards some diurnal, conspicuous groups like butterflies (Ovaskainen 2004), dragonflies (Macagno et al. 2008), and other large, charismatic species (Chiari et al. 2014; Drag et al. 2011). These can be targeted in sufficient numbers by active surveys, whereas fewer insect groups may be targeted by stochastic trapping (Ranius 2001). Judging from a combination of studies of different insects from pests to threatened species, access to pheromonal attractants, or even weaker kairomonal attractants, would increase immensely the potential for performing mark-recapture studies, and consequently, thus obtaining reliable population estimates for many insect species of conservation concern (Creighton and Schnell 1998; Larsson and Svensson 2009; Torres-Vila et al. 2015; Zauli et al. 2014).

## Movement, Dispersal, and Active Range of Traps

Animals are thought to evolve dispersal and colonization strategies in relation to their habitat dynamics (Nilsson and Baranowski 1997; Travis and Dytham 1999). Movement and dispersal are crucial for long-term persistence of most species, to counteract the effects of local extinctions and loss of genetic diversity. The efficiency of ecosystem services such as natural enemies and pollinators in habitats and landscapes is dependent on the mobility of these insects and their interactions with underlying landscape features (Schellhorn et al. 2014). Understanding movement patterns and dispersal biology of insects is thus of major importance for their conservation, especially in landscapes that have been altered significantly by human activity, which are often heavily modified and fragmented to a degree for which most organisms lack adaptations (Ranius 2006; Thomas 2000).

As with population estimates, studies of insect dispersal are heavily skewed towards model systems that allow systematic recapture of individuals. Apart from semiochemical attractants, which mostly have been restricted to pest species (see below), common insect groups targeted in a relevant context again include diurnal and visually conspicuous insects (Ovaskainen 2004; Samways and Lu 2007), and occasionally ground beetles and other insects that may be trapped with sufficient recapture rates (Allema et al. 2014; Elek et al. 2014; Martay et al. 2014; Ranius and Hedin 2001). In addition, insect movement and dispersal have been studied by radio telemetry in relatively large insects (Hedin et al. 2008; Rink and Sinsch 2007; Svensson et al. 2011; Vinatier et al. 2010), and by means of harmonic radar with transponders (Martay et al. 2014; Ovaskainen et al. 2008).

Most studies of insect movement and dispersal by recapture in semiochemical-baited traps have been done with pest insects. These studies include characterization of recapture rates at different distances from traps (see above), or investigation of the landscape-based mobility of pest species in relation to area-wide pest management (Kishita et al. 2003; Yamamura et al. 2003). Nevertheless, the experimental designs and mathematical models developed for these systems likely have general application to studies of patterns of insect movement in response to pheromones.

Pheromone trapping could significantly improve our ability to detect movement of many species and guilds of rare and threatened insects by drastically increasing the probability of capture, and by using systematic capture-recapture points to provide comparable estimates of movement distributions between different model systems. For example, carrion-baited traps obtained recaptures of the carrion beetle *Nicrophorus americanus* over distances of several kilometers (Creighton and Schnell 1998). In contrast, for the scarab beetle *O. eremita*, combined observations from pitfall trapping, pheromone monitoring, and telemetry studies have demonstrated short average dispersal distances in Sweden, with 500 meters as the longest dispersal distance ever observed (Hedin et al. 2008; Ranius and Hedin 2001; Svensson et al. 2011). However, in Central and Southern Europe, this species is more prone to dispersal, with individuals, and especially females, dispersing over distances of hundreds of meters to over 1 km (Chiari et al. 2013a; Dubois and Vignon 2008; Zauli et al. 2014). In contrast, recaptures of *E. ferrugineus* using sex pheromone-baited traps have revealed similar dispersal distances in Sweden and Italy, with the longest observed dispersal distances frequently above 1 km, with flight distances possibly underestimated because of the spacing between traps rather than actual flight capability (M.C. Larsson and G.P. Svensson, unpublished observations from Svensson et al. (2012) and other studies; Zauli et al. 2014).

**Fig. 3:**
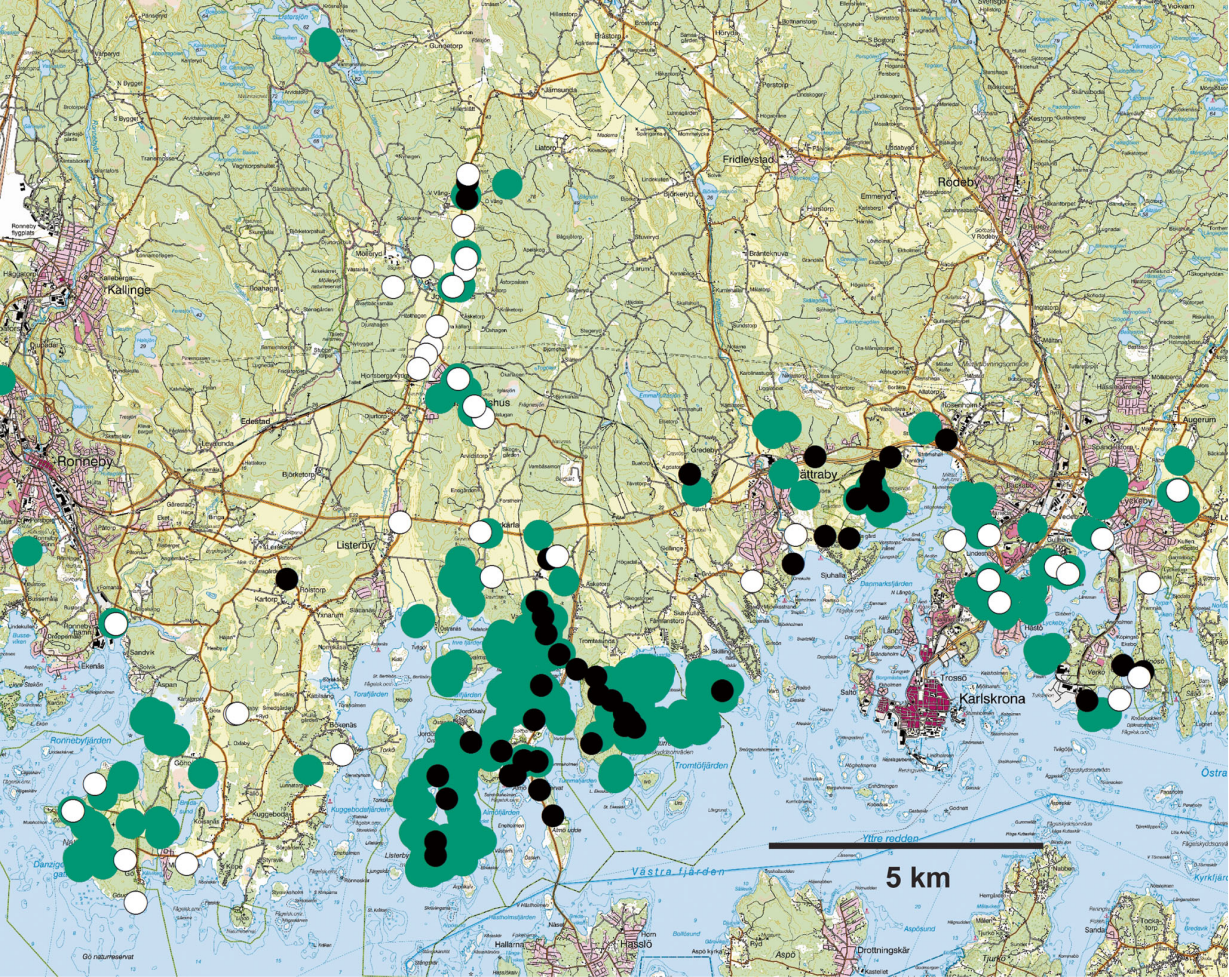
Illustration of “gaps” in the local distribution of the click beetle *Elater ferrugineus* based on pheromone trapping in the Blekinge archipelago in southern Sweden. Green dots represent records of the scarab *Osmoderma eremita* from many different surveys, which define sites with hollow tree habitats that would be principally suitable for *E. ferrugineus*. White dots represent pheromone traps that did not catch *E. ferrugineus*, whereas black dots represent pheromone traps that caught at least one specimen. Several of the sites around the main occupied sites appear to be in principle suitable habitats, yet are unoccupied, suggesting that the local distribution of the species could be limited by dispersal ability (M.C. Larsson, G.P. Svensson, and coworkers, unpublished data). Maps: Terrängkartan **©** Lantmäteriet

One potential problem with using sex pheromone-baited traps to study dispersal by mark-recapture is that they predominantly trap only one sex, usually males. If the sexes differ greatly in their dispersal patterns, collecting information from only males may provide misleading information regarding the colonization ability of populations in a landscape context, because females constitute the limiting sex for establishing new reproductive populations. The possibility of performing area-wide presence-absence studies with pheromones may nevertheless provide an indirect means of demonstrating the limits of dispersal ability of a species, based on their absence from ostensibly suitable habitat patches in the landscape matrix. For at least two species where we have trapped only males with species-specific sex pheromones (*E. ferrugineus*, Fig. 3; M.C. Larsson, G.P. Svensson and coworkers, unpublished data, and the longhorn beetle *Prionus coriarius*; I. Winde and coworkers, unpublished data), we frequently have found empty, yet apparently suitable habitat patches near occupied sites, which strongly suggests a limited dispersal ability of the species at the landscape level. For the latter species, this may be due in part to the differences in the propensities of the sexes to fly. Similarly, Ray et al. (2014) have demonstrated with pheromone traps that the threatened longhorn beetle *Desmocerus californicus dimorphus* is almost certainly absent from some restored habitat patches, suggesting long lag phases before recolonization.

It also must be mentioned that the presence of attractive traps may constitute a potential confounding effect on the dispersal behavior of the insects under study (Yamamura et al. 2003). That is, traps may arrest dispersal, or conversely, could in theory attract individuals from greater distances than they would normally traverse. Ideally, from the perspective of studies of movement patterns, the range of active attraction of a trap should be a small fraction of the normal dispersal range of the species. The true attractive range of a trap is difficult to estimate because it is hard to know how much of the distance between release and recapture sites constitutes random dispersal vs. active movement towards the source, respectively (Byers 2008; Byers et al. 1989). Estimates from behavioral observations or capture experiments around a pheromone source vary widely from tens to a few hundred meters among insect species and lure systems (Linn et al. 1987; Östrand et al. 2000; Sufyan et al. 2011), but sustained attraction over only tens of meters appears more frequent than anecdotal reports about attraction over vast distances. In studies of dispersal between sites that are situated relatively far away from each other, most observed dispersals probably constitute genuine dispersal events, with the lure being effective at close range, rather than actively causing dispersal by long-range attraction to a pheromone lure. If active attraction to pheromone-baited traps is expected to seriously affect movement patterns of the target insects, trapping should be limited to discrete periods interspersed with trap-free periods to allow the insects to redistribute between trapping events.

## Landscape and Habitat Interactions

A fundamental goal of most conservation efforts is to provide sufficient suitable resources available at the landscape scale to ensure long-term persistence of populations. This may include specific habitats, food sources, host plants, or dead-wood substrates at the right stage of decomposition. The core question that conservation science has to answer concerns the amount of provided resources that are indeed sufficient to sustain populations of target species at both different spatial and temporal scales (Fahrig 2001; Holland et al. 2004). Evidence-based answers to these questions often are obtained by relating the distribution of target species to various amounts of resources, habitat types, and other landscape parameters (Buse et al. 2007; Ranius and Nilsson 1997), or in relation to specific landscape or habitat management measures (Collins et al. 1998; Görn and Fischer 2015). As detailed above, however, the difficulty in obtaining accurate data on distribution and abundance could throw these estimates off by a wide margin. Large-scale trapping with pheromones or other semiochemicals provides a standardized way of simultaneously sampling a large number of potential habitats, along broad gradients of differing landscape variables, with considerable accuracy and minimal effort (Gandhi et al. 2009; Jurzenski et al. 2014; Musa et al. 2013; Schroeder 2013). Whereas passive sampling methods rely to a great extent on hotspots or substrate elements where insects aggregate (Brunet and Isacsson 2009), semiochemically-baited traps can be dispersed systematically in the landscape to provide a measure of abundance that is independent of the underlying habitat structure (Benedick et al. 2006; Hanski et al. 2007; Jurzenski et al. 2014; Musa et al. 2013).

The need for highly attractive traps also is dependent on the questions being asked and the model systems available. For example, in Gandhi et al. (2008), stochastic pitfall trapping was sufficient to characterize general changes among populations of common ground beetle species in response to catastrophic wind disturbance and contrasting management techniques in forest stands. Conversely, describing the overall responses of common subcortical saproxylic insects to the same disturbance events depended largely on traps baited with a series of broad-spectrum pheromone-kairomone blends (Gandhi et al. 2009).

**Fig. 4:**
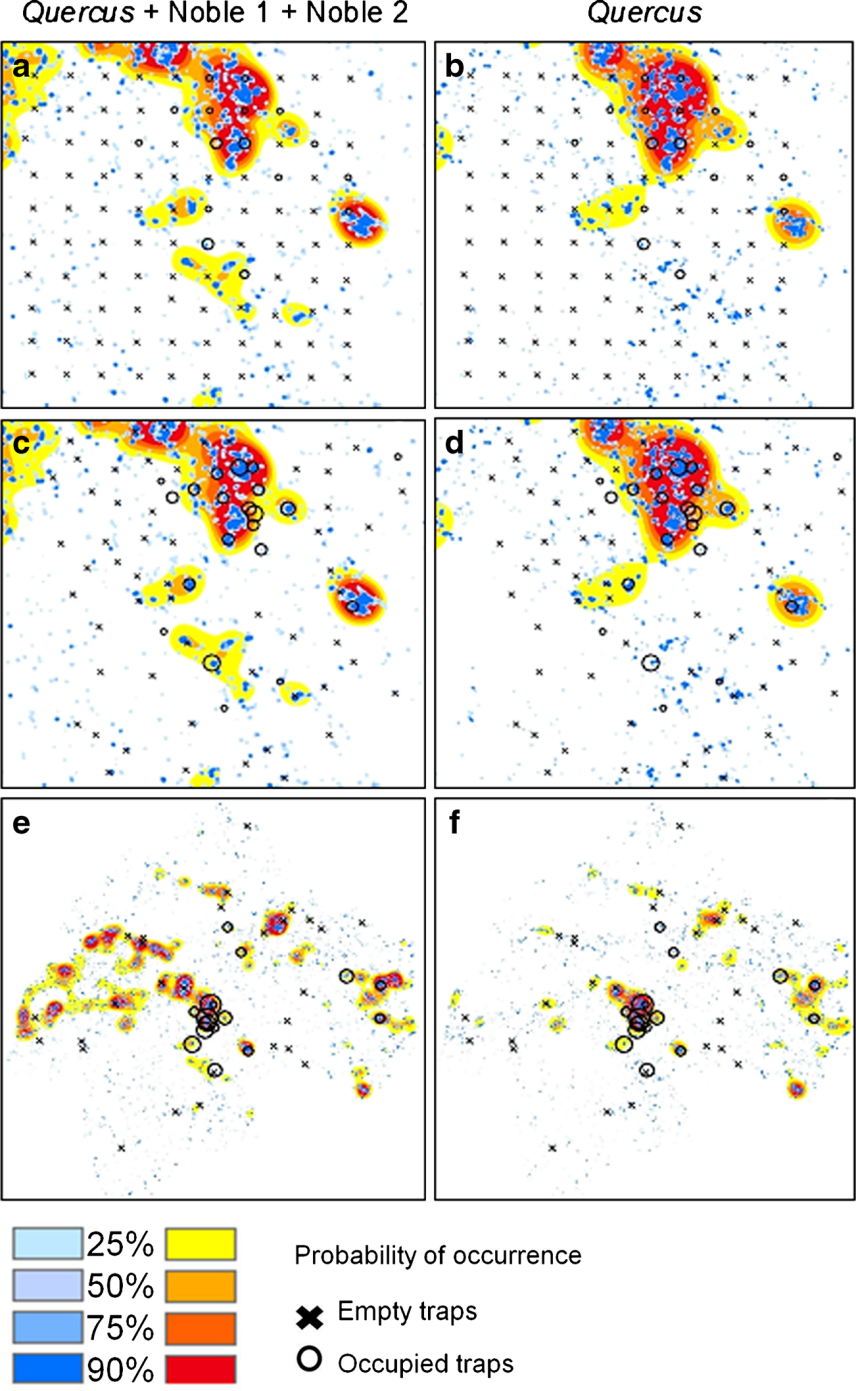
Quantitative landscape models predicting the presence of the saproxylic click beetle *Elater ferrugineus* at >25%, >50%, >75% and >90% probability of occurrence in relation to the amount of habitat (mature trees) in the landscape. Trap catches and predictive models in the model study area (**a,b,c,d**) and in the county of Östergötland (**e,f**). Empty traps are represented by crosses (×) while occupied traps are marked with open circles (O) whose sizes are proportional to the number of individuals caught. The first column (**a,c,e**) shows predictions from pooled density of oak (*Quercus*) and other ´noble´ hardwoods, while the second column (**b,d,f**) is based on the density of *Quercus* only. (**a,b**) shows trap captures in systematically placed traps used to generate the model, (**c,d**) shows traps used for strategically sampled validation data, (**e,f**) represents a validation data set sampled over the entire Östergötland county. In each map, the predictions are based on two models, one for each characteristic scale of best response (blue tones represent a smaller scale: 433 m (pooled density of *Quercus*, Noble 1 and Noble 2) and 327 m (density of *Quercus* only), while orange tones represent predictions at larger scale: 4051 m (*Quercus*, Noble 1 and Noble 2) and 4658 m (*Quercus*). Figure from (Musa et al. 2013)

In contrast, accurately describing the distribution of individual rare species in relation to specific landscape features represents a formidable challenge of an entirely different magnitude, for which highly efficient large-scale trapping systems may provide a distinct advantage (Burman et al. 2016; Kadej et al. 2015; Musa et al. 2013; Oleksa et al. 2015) (D. Harvey and coworkers, unpublished data). To date, the most illustrative of the few examples available may be our single non-destructive survey of the click beetle *E. ferrugineus* across hundreds of sites in southeastern Sweden, which more than doubled the number of known sites, with the result that the distribution of this species now ranks among the best known of the Swedish insect fauna (Andersson et al. 2014; Forsmark 2012; Musa et al. 2013) (J. Burman and coworkers, unpublished data). A focused effort with more than 200 trap sites in the county of Östergötland allowed sampling of abundance in relation to the density of hollow trees surveyed across the whole county, followed by the generation and testing of predictive models for critical habitat abundance thresholds (Musa et al. 2013) (Fig. 4). This single effort thus improved on decades of information gathering (Ranius et al. 2011).

## Indicator Potential of Model Species

As discussed above, comprehensive characterization of landscape variables or biodiversity via broad surveys is time consuming and expensive. Therefore, it is common to use proxy values in the form of bioindicators, that is, limited selections of species or groups of organisms that are believed to provide general information about important variables for conservation planning (Lewandowski et al. 2010; Lindenmayer et al. 2000; McGeoch 1998). Some species that are especially charismatic and/or believed to be of special significance for certain habitats may be designated umbrella or flagship species, and thus constitute both public symbols and practical indicators for the conservation of whole communities of organisms (Lambeck 1997). *Osmoderma eremita* is designated as an umbrella species for giant oak habitats, under the EC/EU Habitats Directive (Anonymous 1992). The conservation value of both the indicator and umbrella species concepts have been questioned (Andelman and Fagan 2000), but nevertheless have evidentiary support and remain central tools in practical conservation.

It is obvious that pheromone-based trapping systems would be an ideal tool for frequent monitoring of limited numbers of indicator species, provided that suitable insect species could be found for a given conservation target. A number of saproxylic beetles dependent on old, mature trees have been suggested as suitable indicators for the continuity of mature trees and old-growth forest (Nilsson et al. 1995; Nilsson and Baranowski 1994; Ranius 2002). Unfortunately, many of these species are themselves difficult to detect and/or would require invasive sampling techniques, and so have been considered unsuitable for practical use as indicators. However, many are known or expected to use long-range pheromones, and pheromone-based monitoring would transform sampling them from a formidable specialist endeavor to an almost trivial task. Again, to date, this type of transformation of monitoring effort is perhaps best exemplified by the click beetle *E. ferrugineus*, which recently was confirmed to have high potential as an indicator for threatened saproxylic fauna of mature trees entirely based on pheromone monitoring (Andersson et al. 2014).

However, the specific relationship between various indicator species and other insect fauna of hollow trees appears to be highly dependent on region (Jansson et al. 2009). In woodlands of both the Mediterranean (Zauli et al. 2014) and the UK (D. Harvey and coworkers unpublished data), the occupancy pattern of *E. ferrugineus* in different habitats and in relation to other saproxylic insects appears to differ considerably from that of Swedish populations. Nevertheless, it is likely to constitute a valuable indicator of mature tree faunal continuity across the species’ European range. More generally, not all rare and endangered species constitute relevant indicator species, even when they can be monitored efficiently with pheromones. For example, large-scale pheromone surveys of the clearwing moth *Synanthedon vespiformis*, which is red-listed as Vulnerable in Sweden, revealed a scattered distribution with no apparent correlation with the local abundance of old oaks, which constitute its nominal habitat (Burman et al. 2016).

## More Harm Than Good?

Pheromone monitoring of threatened insect species entails one specific concern that is uniquely different from monitoring of pest insects, namely whether trapping could harm the target population. When dealing with endangered insects, which often have limited distributions and sometimes exist at very low population densities, the potential for accidental population extinctions becomes a real concern. Even in cases with negligible risk to local populations, the prospect of killing substantial numbers of rare insects in the process of monitoring them may appear distasteful to many, and it certainly will not facilitate general acceptance or the process of obtaining necessary permits. Fortunately, most insect species can be trapped live with appropriate trap designs and released, and these sorts of traps certainly can be used with sensitive species or populations. Nevertheless, opting for killing traps may avoid many problems with inefficient retention of trapped insects and associated risks of unreliable quantitative data. Most importantly, the full potential of pheromone trapping systems for large-scale surveys will be realized only if traps can be deployed for long durations, without the need for frequent and costly visits to release captured specimens.

In reality, most insect populations would likely be more at risk of extinction from failure to act on good information than from pheromone-based trapping. Knowledge of their existence is a prerequisite to prevent populations from going extinct due to habitat exploitation and destruction, or simply due to failures to implement appropriate land management (Balmer and Erhardt 2000; Bengtsson et al. 2000; Olff et al. 1999). Unfortunately, legal or bureaucratic procedures in the United States and in some countries in Europe and many other parts of the world, appear overly restrictive when it comes to protecting even individual insect specimens of certain species or at certain sites, without considering the actual risk to local populations and the value of research for their preservation. Pheromone trapping systems usually are efficient enough to allow non-destructive sampling with sufficient statistical power to generate reliable data on population densities. The possibility of obtaining scientifically-based population estimates should provide additional arguments that insect populations generally are not being harmed by careful sampling.

Based on the capture rates observed in most pheromone trapping systems (see above), low-density trapping should be effective for detecting populations, but should not capture enough individuals to harm the population. The risk can be further limited by trapping during only a fraction of the expected activity period of the target species. The most efficient pheromone trapping systems appear to be those that use sex pheromones to attract males, which are rarely in short supply due to the operational sex ratio, whereas pheromones attracting the more critical females generally are less effective. Decades of pest management have demonstrated that eradication and population control by means of mass trapping with pheromones generally requires sustained, intensive efforts (El-Sayed et al. 2006), suggesting that low numbers of traps deployed for limited time periods should pose little risk to most insect populations. On the other hand, rare and threatened insects differ from pest insects in their overall lower population densities, which may sometimes put them in the range where even widely spaced traps could result in inadvertent mass trapping, in combination with other risk factors such as Allee effects (El-Sayed et al. 2006; Liebhold et al. 2016). In order to deploy pheromone monitoring systems for conservation with maximum efficiency and minimum risk, it would be important to document capture rates and other important characteristics, and evaluate their potential for other negative effects on the target insects (Oleander et al. 2015).

## Conclusions

Monitoring of rare and threatened insects based on exploitation of pheromones or other semiochemicals has the potential to revolutionize the conservation of many insect groups. Pheromone-baited traps could vastly improve our ability to monitor specific species with unprecedented spatiotemporal resolution, with minimum effort and limited risk to target populations. They would provide an excellent means of identifying biodiversity hotspots, tracking population changes, identifying habitat thresholds for persistence of target species at the landscape level, and providing feedback to evaluate the effects of conservation management efforts. Thus far, this potential has only been realized to a limited extent. Further incorporation of pheromone-based monitoring systems into mainstream conservation biology will require development of model systems for strategic species, and further study of their operational characteristics and their ability to provide relevant information.
